# Analysis of retrograde infection of prophylactic pelvic drains in rectal cancer surgery: a retrospective cohort study

**DOI:** 10.1007/s00595-026-03273-5

**Published:** 2026-03-19

**Authors:** Yuzo Nagai, Hiroaki Nozawa, Kazuhito Sasaki, Koji Murono, Shigenobu Emoto, Kensuke Kaneko, Yuichiro Yokoyama, Shinya Abe, Yuzo Harada, Takahide Shinagawa, Yuichi Tachikawa, Soichiro Ishihara

**Affiliations:** Department of Surgical Oncology, Faculty of Medicine, The University of Tokyo, 7-3-1 Hongo, Bunkyo-ku, Tokyo, 113-0033 Japan

**Keywords:** Prophylactic drain, Drain infection, Surgical site infection, Rectal cancer, Surgery

## Abstract

**Purpose:**

The role of prophylactic pelvic drains in rectal surgery remains controversial. This study aimed to clarify the clinical impact of one major concern: retrograde drain infection.

**Methods:**

The subjects of this retrospective study were 852 consecutive patients who underwent rectal cancer resection with bowel anastomosis and drain placement at a single referral hospital in Japan. Retrograde drain infections were categorized based on infection depth, as superficial/deep or organ/space. Patients with anastomotic leakage (*n* = 10) were excluded from the analysis. Outcomes included infection frequency, characteristics, risk factors, and oncological effects.

**Results:**

Retrograde drain infection developed in 5.5% of the patients, with the vast majority (84.8%) diagnosed on or after postoperative day (POD) 7. Male sex was a significant risk factor (7.0% vs. 3.3%, *P* = 0.025). Patients with a retrograde drain infection had longer hospital stays (median 22 vs. 16 days, *P* < 0.001). Organ/space infections were associated with poorer recurrence-free survival for patients with Stage I–III disease. Drain placement helped early detection in 6 of 10 patients with anastomotic leakage, avoiding stoma creation in 2 patients.

**Conclusion:**

Retrograde drain infection occurs more frequently in male patients and prolongs the hospital stay. While prophylactic drains may aid early leakage detection, removal within 7 days is recommended to reduce infection risk.

**Supplementary Information:**

The online version contains supplementary material available at 10.1007/s00595-026-03273-5.

## Introduction

No clear consensus has been reached about the benefits and risks of prophylactic pelvic drain placement in rectal surgery. Several randomized trials and meta-analyses have suggested that drains provide no clinical benefit, with outcomes such as pelvic sepsis, anastomotic leakage, surgical morbidity, reoperation rates, and hospital stay being comparable between patients with and those without drains. Moreover, drain placement may increase the risks of complications such as postoperative ileus and small-bowel obstruction [1–4].

Despite ongoing debate, prophylactic pelvic drains offer advantages for selected patients. By facilitating fluid and blood drainage, they may help prevent the development of abscesses or hematomas. More importantly, they enable early detection of major postoperative complications such as anastomotic leakage or intraabdominal bleeding. Some studies have also reported lower reoperation rates and postoperative mortality in patients with pelvic drains [5–7]. In some cases, anastomotic leakage can be treated conservatively with drains, avoiding invasive interventions such as reanastomosis or stoma creation [8, 9]. Based on these potential benefits, prophylactic pelvic drains are commonly placed after middle and low rectal cancer surgery in our department.

A major concern of prophylactic drain placement is retrograde drain infection [4, 10]. This is a type of surgical site infection (SSI) that occurs at the drain insertion site. Pathogens may migrate through this site, leading to infections in the skin, subcutaneous tissue, muscle, or abdominal cavity. SSIs are associated with higher mortality rates, longer hospital stays, increased medical expenses, and negative cancer-related outcomes, underscoring the need for preventive strategies [11, 12]. Despite its clinical importance, retrograde drain infection is not well understood. We conducted this study to evaluate the incidence, characteristics, risk factors, and oncological impact of retrograde drain infection in patients undergoing rectal resection with bowel anastomosis.

## Methods

### Patients

We reviewed 852 consecutive patients with rectal cancer, who underwent rectal resection with bowel anastomosis and prophylactic pelvic drain placement at our institution between January, 2010 and December, 2022. Clinical, surgical, and pathological data were collected from the medical records. Cancer staging followed the TNM Classification of Malignant Tumor (8th Edition) [13]. Preoperative chemoradiotherapy (CRT) with long-course radiation and 5-fluorouracil-based chemotherapy was indicated for patients with clinical stage T3 or higher lower rectal cancer. Lateral pelvic lymph node dissection was performed in patients with clinically suspected metastasis. The colo-anal anastomosis was done using either the intracorporeal double stapling technique (DST) or a hand-sewn anastomosis, based on the tumor location. A protective diverting stoma was created when indicated. Patients who underwent abdominoperineal resection and total pelvic exenteration were excluded from the analysis, as these procedures involve perineal wounds and specific postoperative complications that could have confounded the assessment of retrograde drain infection.

A 24-Fr SILASCON duple drain (Kaneka Medical Products, Osaka, Japan) was placed in the pelvic cavity and connected to a closed sterile drainage system. The same drain type and size were used consistently throughout the study period, without any variation among patients. The drain insertion site was covered with a sterile film dressing. Prophylactic second-generation cephalosporin antibiotics were given routinely before skin incision until postoperative day (POD) 1, and this regimen remained unchanged throughout the study period. In the 2010s, our institution maintained pelvic drains for at least 7 days because of concerns about anastomotic leakage. In recent years, the standard practice has been to remove drains within 7 days following surgery.

### Diagnosis of retrograde drain infection

**Fig. 1:**
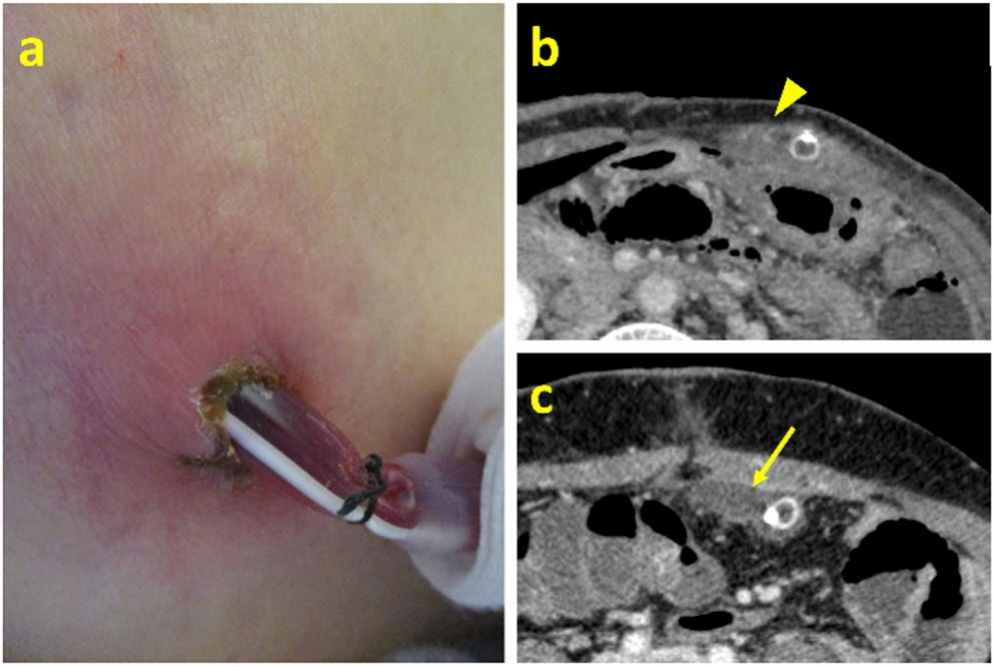
Representative findings of retrograde drain infection. (**a**) Photograph of the drain insertion site showing redness and swelling. (**b**) Computed tomography (CT) scan image showing inflammation of the soft tissue and muscle around the drain, classified as deep surgical site infection (SSI) (arrowhead). (**c**) CT scan image showing intraabdominal inflammation and fluid collection along the drain insertion route, classified as organ/space SSI (arrow)

As no standardized definition exists, for this study, retrograde drain infection was defined as infections detected at the drain insertion site or along the drain insertion route. Diagnosis was based on the following characteristics: pain, tenderness, redness, and swelling around the drain insertion site; purulent discharge from the drain insertion site; and computed tomography (CT) evidence of inflammation of the skin, subcutaneous tissue, muscle, or abdominal cavity along the drain insertion route. CT was not done routinely; instead, imaging was used selectively in patients presenting with severe symptoms or when differential diagnosis such as anastomotic leakage was clinically required. Figure 1 shows the representative findings.

According to the SSI criteria of the Centers for Disease Control and Prevention National Healthcare Safety Network [14], we classified retrograde drain infections into two categories: “superficial/deep” and “organ/space,” based on the depth of involvement. This classification has been used widely in recent surgical literature to evaluate clinical outcomes [15, 16].

### Statistical analysis

Ten patients with anastomotic leakage were excluded from the final analysis of retrograde drain infection, as infections in these patients could not be distinguished from secondary infections related to leakage. Continuous variables are expressed as the median [interquartile range (IQR)] and analyzed using the Mann–Whitney U test for two groups or the Kruskal–Wallis test for multiple groups. Categorical variables are presented as numbers (%) and analyzed using Pearson’s chi-square test or Fisher’s exact test as appropriate. Kaplan–Meier survival analysis was used to assess survival outcomes, and survival differences between groups were evaluated by the log-rank test. Survival outcomes were defined as follows [17]: overall survival (OS), time from surgery to death from any cause; recurrence-free survival (RFS), and the time from surgery to cancer recurrence. To identify the independent risk factors for retrograde drain infection, we constructed a multivariable logistic regression model including sex, body mass index, diabetes mellitus, operative time, and surgical period, as covariates. These variables were selected a priori based on their clinical relevance rather than based on univariate significance. To assess whether retrograde drain infection was independently associated with postoperative hospital stay, we also constructed a multivariable linear regression model including retrograde drain infection, age, sex, operative time, surgical approach, diverting stoma, and surgical period as covariates. A *P* value of 0.05 was considered significant. Statistical analyses were carried out using JMP pro version 14 software package (SAS Institute, Cary, NC, USA).

## Results

### Incidence and characteristics of retrograde drain infection

**Fig. 2:**
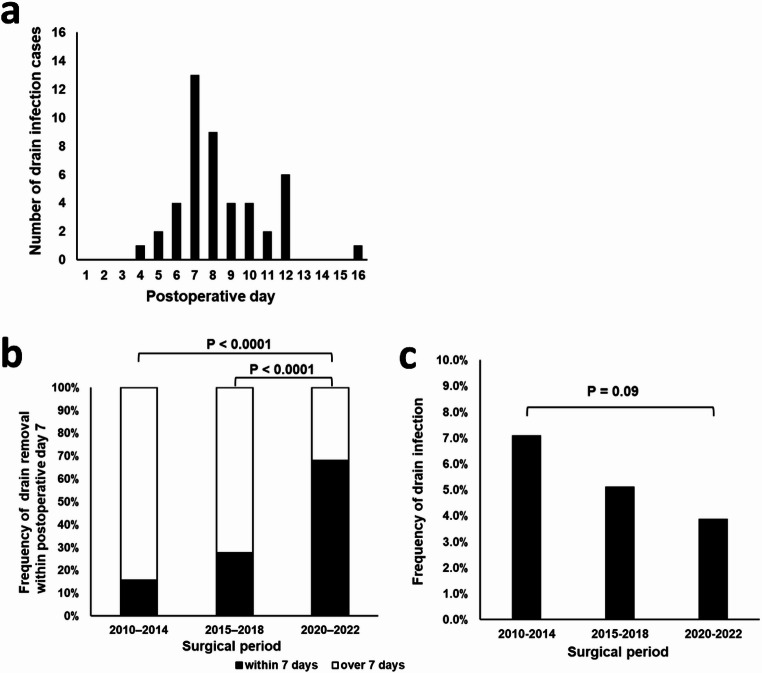
Timing of retrograde drain infection onset and its relationship with drain duration and infection frequency across the surgical periods in this study. (**a**) 84.8% (39/46) of drain infections were diagnosed on postoperative day (POD) 7 or later. (**b**) The percentage of patients with drains removed within 7 days increased significantly over time. (2010–2014: 15.7%, 2015–2018: 27.7%, 2020–2022: 68.0%) (**c**) The retrograde infection rate tended to decrease accordingly (2010–2014: 7.1%, 2015–2018: 5.1%, 2020–2022: 3.9%)

Table 1 presents an overview of retrograde drain infection. Retrograde drain infection developed in 5.5% (46/842) of the patients. The infection manifested as elevated inflammatory responses, with a median white blood cell count (WBC) of 10,300/µL (IQR 8100–12100/µL) and a median C-reactive protein (CRP) level of 9.1 mg/dL (IQR 5.6–14.1 mg/dL). Common clinical findings included fever (35/46, 76.1%), redness or swelling (34/46, 73.9%), pain or tenderness (31/46, 67.4%), and purulent discharge (30/46, 65.2%). All these patients were treated with antibiotic therapy and there was no mortality. The infection depth was superficial/deep in 30 patients and organ/space in 16. Bacterial cultures were performed in 25 of these 46 patients. All detected bacteria were skin commensals, with methicillin-susceptible *Staphylococcus aureus* (32.6%) being the most common, followed by *Pseudomonas aeruginosa* (15.2%) and methicillin-resistant *S. aureus (MRSA)* (6.5%). Most (84.8%; 39/46) of the drain infections were diagnosed on POD 7 or later (Fig. 2a). Over the years, earlier drain removal within 7 days has become more common (2010–2014: 15.7%, 2015–2018: 27.7%, 2020–2022: 68.0%) (Fig. 2b), and infection rates have decreased accordingly (2010–2014: 7.1%, 2015–2018: 5.1%, 2020–2022: 3.9%) (Fig. 2c). This suggests that prolonged drain placement increases infection risk.

**Table 1:** Overview of retrograde drain infection (*n* = 46)

Overall incidence of retrograde drain infection, *n* (%)	46/842 (5.5)
WBC, median (IQR)	10,300 (8100–12,100)
CRP, median (IQR)	9.1 (5.6–14.1)
Clinical signs and symptoms	
Fever, n (%)	35 (76.1)
Redness or swelling at the drain site, n (%)	34 (73.9)
Pain or tenderness at the drain site, n (%)	31 (67.4)
Purulent discharge from the drain site, n (%)	30 (65.2)
The depth of infection	
Superficial/ deep, n (%)	30 (65.2)
Organ/ Space, n (%)	16 (24.8)
Results of bacterial culture	
Methicillin-susceptible Staphylococcus aureus, n (%)	15 (32.6)
Methicillin-resistant Staphylococcus aureus, n (%)	3 (6.5)
Pseudomonas aeruginosa, n (%)	7 (15.2)
Bacterial culture not performed, n (%)	21 (45.7)

WBC, white blood cell count; IQR, interquartile range; CRP, C-reactive protein

### Risk factors for retrograde drain infection

To assess the factors influencing retrograde drain infection, clinical, tumor, and surgical factors were compared between patients with and those without infection (Table 2). Male patients had a significantly higher infection rate than female patients (6.9% [35/509] vs. 3.3% [11/333], *P* = 0.026). However, no significant differences were found in body mass index, preoperative therapy, history of abdominal surgery, smoking, steroid use, or comorbidities. There were also no significant differences in tumor-related factors, including distance from the anal verge, tumor stenosis, and pathological stage. Surgical factors such as bowel preparation, surgical approach, anastomosis type, operative time, bleeding, intraoperative blood transfusion, rate of combined resection of other organs, presence of diverting stoma, rate of lateral pelvic lymph node dissection, and the incidence of a positive radial margin did not differ significantly.

**Table 2:** Comparison of clinical, tumor, and surgical factors between patients with vs. those without retrograde drain infection

Factors	With drain infection (*n* = 46)	Without drain infection (*n* = 796)	*P* value
Clinical factors			
Age, years, median (IQR)	63 (54–73)	64 (55–72)	0.97
Sex, n (%)			0.026
Male	35 (76.1)	474 (59.6)	
Female	11 (23.9)	322 (40.4)	
BMI (kg/m^2^), median (IQR)	22.8 (20.3–25.3)	22.6 (20.2–24.8)	0.53
Preoperative therapy, n (%)			0.58
Chemoradiotherapy or radiotherapy	11 (23.9)	220 (27.6)	
None	35 (76.1)	576 (72.4)	
History of abdominal surgery, n (%)	11 (23.9)	241 (30.3)	0.36
Current smoking, n (%)	7 (15.2)	151 (19.0)	0.53
Current steroid use, n (%)	0 (0.0)	12 (1.5)	0.40
Diabetes mellites, n (%)	8 (17.4)	140 (17.6)	0.97
HbA1c (%)			0.98
<7.0%	43 (93.5)	745 (93.6)	
≥7.0%	3 (6.5)	51 (6.4)	
Cardiovascular disease, n (%)	4 (8.7)	89 (11.2)	0.60
Pulmonary disease, n (%)	7 (15.2)	153 (19.3)	0.50
Liver disease, n (%)	4 (8.7)	33 (4.2)	0.14
Tumor factors			
Tumor distance from anal verge (cm), median (IQR)	9 (5–13)	8 (5–11)	0.25
Tumor stenosis, n (%)	5 (10.9)	102 (12.8)	0.70
Pathological T stage			0.15
pCR, n (%)	3 (6.5)	29 (3.6)	
T1–T2, n (%)	24 (52.2)	327 (41.1)	
T3–T4, n (%)	19 (41.3)	440 (55.3)	
Pathological tumor stage			0.16
0–II, n (%)	33 (71.7)	467 (58.7)	
III, n (%)	9 (19.6)	261 (32.8)	
IV, n (%)	4 (8.7)	68 (8.5)	
Surgical factors			
Mechanical bowel preparation, n (%)	42 (91.3)	713 (89.6)	0.71
Chemical bowel preparation, n (%)	42 (91.3)	762 (95.7)	0.16
Surgical approach			0.26
Laparotomy, n (%)	3 (6.5)	96 (12.1)	
Laparoscopy or robot-assisted, n (%)	43 (93.5)	700 (88.0)	
Anastomosis			0.89
DST, n (%)	39 (84.8)	669 (84.1)	
Hand-sewn, n (%)	7 (15.2)	127 (15.9)	
Operative time (min), median (IQR)	283 (224–390)	321 (239–431)	0.11
Operative bleeding (ml), median (IQR)	40 (5–165)	40 (10–160)	0.76
Intraoperative blood transfusion, n (%)	0 (0.0)	22 (2.8)	0.25
Combined resection of other organs, n (%)	5 (10.9)	75 (9.4)	0.74
Diverting stoma, n (%)	18 (39.1)	332 (41.7)	0.73
Lateral pelvic lymph node dissection, n (%)	2 (4.4)	106 (13.3)	0.08
Positive radial margin	1 (2.6)	18 (2.3)	0.97

IQR, interquartile range; BMI, body mass index; pCR, pathological complete response; DST, double stapling technique

Patients with anastomotic leakage (*n* = 10) were excluded from the analysis

To evaluate the independent risk factors further, a multivariable logistic regression analysis was performed using five prespecified variables (sex, body mass index, diabetes, operative time, and surgical period), selected based on clinical relevance. In this model, male sex remained an independent risk factor for retrograde drain infection (adjusted odds ratio 2.22, 95% confidence interval 1.13–4.70, *P* = 0.03), but the other variables were not significantly associated with infection (Table 3).

**Table 3:** Multivariable logistic regression analysis of risk factors for retrograde drain infection

Factors	Adjusted OR (95% CI)	*P* value
Male sex (vs. female)	2.22 (1.13–4.70)	0.03
BMI (per 1 kg/m²)	1.17 (0.56–2.28)	0.66
Diabetes (yes vs. no)	1.05 (0.49–2.52)	0.90
Operative time (per 60 min)	0.95 (0.39–2.02)	0.09
Surgical period 2010–2014 (vs. 2019–2022)	1.62 (0.76–3.64)	0.22
Surgical period 2015–2018 (vs. 2019–2022)	1.21 (0.51–2.94)	0.89

OR, odds ratio; CI, confidence interval; BMI, body mass index

### Impact of the postoperative hospital stay

Patients with retrograde drain infections had significantly longer hospital stays [median 22 (IQR 18–28) vs. 16 (IQR 13–19) days, *P* < 0.001] (Table 4). To identify whether drain infection was independently associated with prolonged hospitalization, we performed a multivariable linear regression analysis adjusting for clinically relevant pre- and intraoperative factors (age, sex, operative time, surgical approach, diverting stoma, and surgical period). The results demonstrated that retrograde drain infection remained an independent factor associated with longer hospital stays (Supplementary Table 1).

**Table 4:** Analysis of the postoperative hospital stay

Postoperative hospital stay, median (IQR)	With drain infection (*n* = 46)			Without drain infection (*n* = 796)	*P* value
	22 (18–28)			16 (13–19)	< 0.001
	Immediate drain removal (*n* = 11)	Delayed drain removal (*n* = 35)	P value		
Postoperative hospital stay, median (IQR)	18 (14–24)	23 (19–30)	0.03		
WBC, median (IQR)	10,300 (7600–13,300)	9800 (7800–12,000)	0.68		
CRP, median (IQR)	6.7 (4.9–9.6)	9.1 (5.6–14.2)	0.22		
Depth of infection			0.50		
Superficial/deep, n (%)	6 (54.5)	23 (65.7)			
Organ/space, n (%)	5 (45.5)	12 (34.3)			

IQR, interquartile range; WBC, white blood cell count; CRP, C-reactive protein

Next, we investigated the relationship between the timing of drain removal and hospital stays in patients with a retrograde drain infection. After the diagnosis of retrograde drain infection, 11 had immediate removal, while 35 had delayed removal (11 of these patients had their drains replaced with a new open drain). Because there are no established guidelines for the management of retrograde drain infection, the decision regarding immediate or delayed removal depended on the clinical judgment of the responsible surgeon. WBC, CRP, and infection depth did not differ significantly, but hospital stay was shorter in the immediate removal group [median 18 (IQR 14–24) vs. 23 (IQR 19–30) days, *P* = 0.03] (Table 4).

### Anastomotic leakage

Postoperative anastomotic leakage developed in 10 patients (1.2%) and was diagnosed within 7 days in 7 of these patients, with 6 cases detected by changes in drain content. Leakage was identified after drain removal in five patients, triggered by fever, abdominal pain, or elevated inflammatory markers. Five patients required emergency surgery, including stoma creation, while five were managed conservatively. Notably, two patients (no. 3 and no. 5) without a diverting stoma at primary surgery were treated successfully with drain management, avoiding stoma creation (Table 5).

**Table 5:** Diagnosis and treatment summary of the 10 patients with anastomotic leakage

Case	Postoperative day of diagnosis	Initial trigger for diagnosis	Postoperative day of drain removal	Diverting stoma at primary surgery	Treatment	Postoperative hospital stay, days
1	0	Changes in drain content	41	No	Emergency colostomy	44
2	4	Changes in drain content	40	Yes	Conservative treatment with drain management	55
3	4	Changes in drain content	42	No	Conservative treatment with drain management	42
4	6	Changes in drain content	30	Yes	Conservative treatment with drain management	42
5	6	Changes in drain content	32	No	Conservative treatment with drain management	90
6	6	Changes in drain content	63	Yes	Emergency intra-abdominal irrigation and drainage	71
7	7	Fever	33	No	Emergency colostomy	37
8	8	Fever and abdominal pain	7	No	Emergency colostomy	36
9	9	Elevated inflammatory response	7	No	Conservative treatment	37
10	19	Fever and abdominal pain	9	No	Emergency ileostomy	54

### Oncological impact

The median follow-up period was 59.7 months for patients with a drain infection and 60.4 months for those without a drain infection. In the prognostic analysis, patients with a retrograde drain infection were stratified into superficial/deep (*n* = 29) and organ/space (*n* = 17) subgroups.

**Fig. 3:**
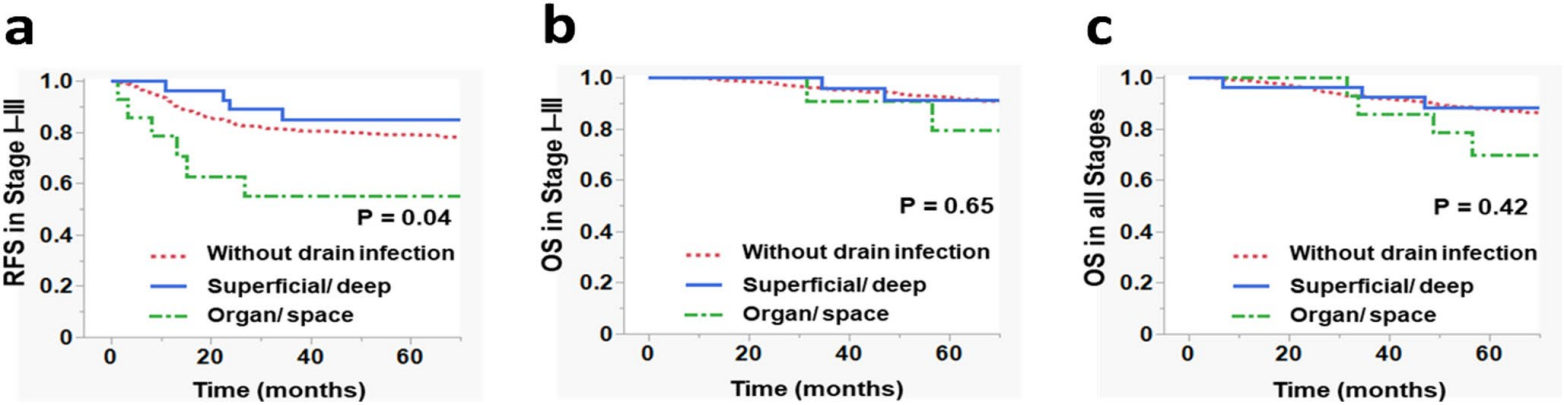
Kaplan–Meier survival curves comparing patients with no drain infection, those with superficial/deep infection, and those with organ/space infection. (**a**) Recurrence-free survival of patients with Stage I–III disease (**b**) Overall survival of patients with Stage I–III disease (**c**) Overall survival irrespective of stage

Supplementary Table 2 summarizes the pathological stage distributions. While the organ/space group tended to include a higher proportion of patients with pStage III disease than the superficial/deep group, the overall distribution was broadly similar to that of the no infection group. The organ/space drain infection group had significantly worse recurrence-free survival (RFS) for stage I–III disease (5-year RFS: 79.2% no infection, 85.0% superficial/deep, 55.0% organ/space, *P* = 0.04) (Fig. 3a). Overall survival (OS) analysis showed no significant differences for stage I–III disease (5-year OS: 92.3% without infection, 91.4% superficial/deep, 79.6% organ/space, *P* = 0.65) or across all stages (5-year OS: 87.7% no infection, 88.3% superficial/deep, 69.8% organ/space, *P* = 0.42) (Fig. 3b and c).

## Discussion

To our knowledge, this is the first comprehensive analysis focusing on retrograde drain infection after rectal cancer surgery. Although the overall value of prophylactic drains remains debated, they can be useful for early diagnosis and conservative management in selected patients [8, 9]. In our cohort, changes in drain content enabled the early detection of anastomotic leakage in six of ten patients, two of whom without diverting stomas were successfully managed conservatively, avoiding stoma creation. Although there were no cases of postoperative intrabdominal bleeding in this study, prophylactic drains may enable early recognition of bleeding and prevent serious outcomes [18]. Thus, prophylactic pelvic drains still have clinical value, particularly for high-risk rectal cancer patients [19–22]. Conversely, retrograde drain infections were associated with prolonged hospital stays, thereby increasing medical costs and patient burden. According to previous reports, prolonged drain placement can lead to complicated infections requiring vancomycin therapy [10]. In our study, MRSA was identified in 6.5% of the cultures. Although infrequent, this finding underscores the risk of drug-resistant pathogens, which may necessitate the use of broad-spectrum antibiotics, potentially and extend hospitalization further.

Our study showed that most retrograde drain infections occurred after POD 7 and that drains removed early were associated with a lower infection rate. This supports a strategy of early drain removal to reduce the risk of infection. Current international guidelines, including the 2018 ERAS Society recommendations and the 2023 joint guidelines from the American Society of Colon and Rectal Surgeons and the Society of American Gastrointestinal and Endoscopic Surgeons, advise against routine intra-abdominal drainage in colorectal surgery [23, 24]. Nevertheless, drains are still used selectively in rectal cancer surgery, particularly in patients at high risk of anastomotic leakage. In our cohort, seven of ten leaks were diagnosed within 7 days, whereas most retrograde infections developed later. This indicates that prolonged drain placement provides limited additional benefit for leak detection, but increases infection risk substantially. Taken together, these findings suggest that removing drains within 7 days may help balance early complication detection with infection prevention, thus promoting earlier recovery. This approach aligns with ERAS goals and reflects current practices in many centers that have implemented early discharge programs.

Univariate analysis identified male sex as a risk factor for retrograde drain infection. Although the underlying mechanism remains unclear, previous studies have also reported a similar trend, with male patients showing a higher incidence of SSI [12, 15, 25]. Anatomical and hormonal factors may help explain this increased risk. Men tend to have greater visceral adiposity and thicker subcutaneous tissue, both of which can impair wound healing and expediate bacterial colonization [12, 15, 26]. Hormonal differences may also play a role: estrogen has been shown to promote anti-inflammatory responses and accelerate wound healing, whereas androgens such as testosterone are associated with delayed healing and increased inflammatory cell infiltration [27]. These factors may contribute to a higher susceptibility to infection in male patients. Considering these anatomical and physiological differences, targeted prevention strategies against SSI and drain infections may be particularly important in men.

Currently, there are no clear guidelines for the management of retrograde drain infection. Reported strategies vary: some surgeons advocate immediate drain removal after diagnosis to eliminate the infection source, whereas others leave the drain in place until the infection subsides with antibiotics. Additional practices include replacing the drain with a new one or withdrawing it in small increments (for example, 2 cm per day). Although our sample size was limited, the findings of the present study suggest that immediate drain removal may help reduce the postoperative hospital stay.

Anastomotic leakage after rectal cancer surgery is well known to worsen prognosis [28, 29]. A meta-analysis in 2020 on colorectal cancer also reported that nonanastomotic SSIs negatively affect OS and cancer-specific survival [11]. In our study, overall survival did not differ significantly according to SSIs or anastomotic leakage; however, patients with stage I-III disease and organ/space retrograde drain infections had significantly poorer recurrence-free survival (RFS), underscoring the importance of preventing infections. Pathological stage tended to be more advanced in the organ/space infection group, which may have contributed to the difference in RFS. Nevertheless, the separation in survival curves suggests a potential oncological impact that warrants further investigation. Given the small number of events and the absence of multivariate analysis, additional large-scale studies are needed to clarify the prognostic impact of retrograde drain infection.

This study has several limitations. First, although film dressings were used intraoperatively to maintain sterility at the drain insertion site, they were often replaced with gauze either when excessive drainage occurred or when infection was suspected. Because of the lack of detailed documentation of these postoperative wound-care practices, potential variations in clinician-dependent management and their evolution over time may have influenced the infection risk. Second, information on drainage output was also lacking. Third, the relatively long hospital stays reflect Japan’s healthcare system, where universal insurance allows for extended hospitalization without financial burden, and cultural preferences emphasize full recovery before discharge. These factors do not align with practices in many other countries. Furthermore, the postoperative recovery pathways and discharge criteria in Japan generally require patients to reach a higher level of functional recovery before discharge compared with those in Western institutions, which contributes to longer baseline hospital stays irrespective of drain infection status. Fourth, because no standardized diagnostic criteria exist for retrograde drain infection, we defined cases based on inflammatory findings around the drain insertion site and corresponding CT findings. Differentiating retrograde drain infection from subclinical anastomotic leakage or intraoperative contamination remains challenging. Although bacterial cultures were done for only about half of the patients, all identified pathogens were skin commensals, supporting the validity of our diagnostic approach. Future prospective studies with standardized diagnostic and management protocols are warranted to strengthen these findings. Finally, this was a retrospective study from a single institution with a relatively small sample size. Further investigation through large, multicenter cohort studies is warranted.

## Conclusions

Male patients appear to be at higher risk for retrograde drain infections, which are associated with prolonged postoperative hospital stays. Drain placement for 7 days or more may increase the infection risk. Although prophylactic drains are generally discouraged after rectal cancer surgery, they may be useful for the early detection of anastomotic leakage in selected patients. Our findings suggest that drain removal within 7 days provides an appropriate balance between early complication detection and infection prevention, thereby promoting earlier recovery.

## Supplementary Information

Below is the link to the electronic supplementary material.

Supplementary Material 1

Supplementary Material 1
