# Supplementary material for: Analysis of retrograde infection of prophylactic pelvic drains in rectal cancer surgery: a retrospective cohort study
Source: Surg Today. 2026 Mar 19;56(8):1608–16. doi: 10.1007/s00595-026-03273-5 (PMC13379415; doi:10.1007/s00595-026-03273-5)
Supplement: Supplementary file 1 — Supplementary Material 1 [file 595_2026_3273_MOESM1_ESM.docx]

| **Supplementary Table 1.** Comparison of the postoperative hospital stay and results of multivariable linear regression analysis | | | | |
| --- | --- | --- | --- | --- |
| Variable | Postoperative hospital stay, median (IQR) | P value (univariate) | Adjusted β (95% CI) | P value (multivariate) |
| Drain infection (yes vs no) | 22 (18–28) vs 16 (13–19) | <0.0001 | +3.38 (+2.3 – +4.4) | <0.0001 |
| Age (per 10 years) | – |  | +0.74 (+0.3 – +1.1) | 0.0002 |
| Male sex (vs female) | 16 (13–19) vs 16 (13–20) | 0.01 | +0.02 (–0.45 – +0.50) | 0.91 |
| Operative time (per 60 min) | – |  | +0.45 (+0.23 – +0.67) | <0.0001 |
| Surgical approach (Lap/Robot vs Open) | 16 (13–19) vs 17 (15–20) | 0.005 | –1.22 (–1.96 – –0.45) | 0.001 |
| Diverting stoma (yes vs no) | 19 (16–23) vs 14 (12–17) | <0.0001 | +2.36 (+1.8 – +2.9) | <0.0001 |
| Surgical period 2010–2014 (vs 2019–2022) | 17 (14–20) vs 14 (11–18) | <0.0001 | +1.01 (+0.37 – +1.64) | 0.003 |
| Surgical period 2015–2018 (vs 2019–2022) | 17 (13–20) vs 14 (11–18) | <0.0001 | +0.71 (+0.01 – +1.41) | 0.047 |
| IQR, interquartile range; CI, confidence interval. | | | | |
