# Supplementary material for: Analysis of retrograde infection of prophylactic pelvic drains in rectal cancer surgery: a retrospective cohort study
Source: Surg Today. 2026 Mar 19;56(8):1608–16. doi: 10.1007/s00595-026-03273-5 (PMC13379415; doi:10.1007/s00595-026-03273-5)
Supplement: Supplementary file 2 — Supplementary Material 1 [file 595_2026_3273_MOESM2_ESM.docx]

| **Supplementary Table 2.** Distribution of pathological stage of patients with vs. those without retrograde drain infection | | | |
| --- | --- | --- | --- |
| Pathological tumor stage | No infection  (n = 796) | Superficial/deep infection (n = 29) | Organ/space infection  (n = 17) |
| I–II, n (%) | 467 (58.7%) | 24 (82.8%) | 9 (52.9%) |
| III, n (%) | 261 (32.8%) | 4 (13.8%) | 5 (29.4%) |
| IV, n (%) | 68 (8.5%) | 1 (3.4%) | 3 (17.7%) |
